# Optimization of Wire Electric Discharge Machining (WEDM) Process Parameters for AISI 1045 Medium Carbon Steel Using Taguchi Design of Experiments

**DOI:** 10.3390/ma15217846

**Published:** 2022-11-07

**Authors:** Uzair Khaleeq uz Zaman, Usman Ahmed Khan, Shahid Aziz, Aamer Ahmed Baqai, Sajid Ullah Butt, Danish Hussain, Ali Siadat, Dong Won Jung

**Affiliations:** 1 College of Electrical and Mechanical Engineering, National University of Sciences and Technology, Rawalpindi 44000, Pakistan; 2 Laboratoire Conception Fabrication Commande, Arts et Metiers ParisTech, Campus de Metz, 57070 Metz, France; 3 Department of Mechanical Engineering, Jeju National University, 102 Jejudaehak-ro, Jeju-Si 63243, Korea

**Keywords:** AISI 1045 medium carbon steel, analysis of variance, material removal rate, optimization, Taguchi design of experiments, wire electric discharge machining

## Abstract

With the growth of the manufacturing industry, the demand for alloy materials with high hardness, toughness, and impact strength has increased. Since products from such alloy materials are extremely difficult to manufacture with high accuracy and reduced surface roughness using traditional machining techniques, wire electric discharge machining can be used to machine such complex parts with more precision. In this case-study-based research, machining factors such as current, pulse-on time, and voltage are studied to determine their effects on the material removal rate for AISI 1045 medium carbon steel. The Taguchi L_9_ orthogonal array is used in the design of experiments for optimization. Statistical techniques such as analysis of variance and signal-to-noise ratio are used to identify the control parameters that matter most in bringing about optimal results. Based on the results, the current is the most crucial control variable in this investigation. Moreover, the maximum material removal rate obtained was 0.7112 mm^3^/min with the obtained optimized values of current (I) = 16 A, voltage (V) = 50 V, and pulse-on time (Ton) = 100 µs.

## 1. Introduction

Economic globalization constantly pushes the manufacturing sector to advanced and innovative transformations through groundbreaking and state-of-the-art technologies. Hence, to remain competitive in today’s marketplace, mechanical components must be manufactured with high accuracy and reliability and in the shortest possible time. Materials science has developed advanced engineering materials such as super alloys, composites, and ceramics. These materials are difficult to machine and often impede smooth machining with traditional machining processes such as turning, milling, drilling, and grinding. As a result, electrochemical machining, ultrasonic machining, wire cutting, wire electrical discharge machining (WEDM), and other non-traditional methods of machining are used on difficult-to-machine materials.

Wire electric discharge machining (WEDM) is a fast-growing and non-conventional advanced manufacturing process used for high-strength materials. Every hard material which is difficult to cut with other conventional methods can be sliced using WEDM [1]. To meet the rapidly increasing need for materials with unique properties in sophisticated professional programs such as aeronautical and medical devices, WEDM is the best option [2]. The WEDM process extracts particles from the surface through thermal erosion [3]. In order to use WEDM, the workpiece and tool must be electrically conductive [4,5]. The wire acts as a cathode, and the work material acts as an anode. There is no physical contact between the workpiece and the wire, thereby allowing sensitive, breakable objects to be fabricated without risking damage [6,7]. Mechanical stresses are also reduced since the tool and workpiece do not interact during machining [8,9]. An electric shock is further created in discharge energy, and electricity spikes at limited intervals are supplied. A release is normally started when the electric field is improved [10,11]. When the spark comes in contact with the dielectric fluid (present between the workpiece and tool), it ionizes and allows the flow of current between the tool and the workpiece, forming an ionization gradient [12]. This then results in a rapid increase in the temperature of the metal, i.e., from 8000 °C to 12,000 °C (occasionally much higher), causing the surface particles of the workpiece to melt rapidly. The gap between the tool and the workpiece is precisely acclimatized to ensure that ionization occurs. If the gap between the workpiece and the tool is not correctly maintained, there will be no spark, leading to no cutting. Therefore, a constant gap of 0.5 mm is maintained between the workpiece and the tool to produce a spark [13,14]. Moreover, dielectric fluid is mostly used in WEDM with sufficiently high dielectric resistance to not only avoid a quick breakdown electrically but also to ionize when electrons collide with its molecules. Furthermore, during sparking, the dielectric fluid should be thermally resistant as well. In such a case, deionized water is used as a dielectric fluid for most of the WEDM processes [15,16]. In order to measure WEDM’s performance, the literature shows the material removal rate (MRR) and surface roughness [17,18] as the key output factors. Similarly, the popular input factors for WEDM are current, voltage, pulse-on time, pulse-off time, wire tension, and wire speed [19]. Over the years, a lot of interest has been invested by researchers in improving the MRR for faster manufacturing. Here, several variables affect the MRR, such as current, voltage, and pulse-on-time. Current is usually determined during the ‘on-time’ of each pulse while the pulse-on-time is determined as the time during which the current is permitted to flow in each cycle [20,21]. Similarly, voltage influences the MRR as well and can be calculated from the spark zone average power during machining. Voltage also influences the amount of overcutting and the gap in the spark [22,23,24,25,26]. Therefore, in fabricating carbon–carbon alloys, the best WEDM machine setup is for input parameters that involve current, voltage, and pulse-on time, which further also impact the electrode wear rate.

To achieve optimum machining in WEDM, it is critical to choose appropriate machining parameters. These parameters are usually chosen via experience, and it may not guarantee optimum or near-optimal machining performance for WEDM. Gupta et al. [27] studied the effects of WEDM machining input parameters such as servo voltage, wire-feed speed, and wire tension, on surface roughness and cutting speed of titanium (Ti-6Al-4V) alloy using response surface methodology (RSM) and analysis of variance (ANOVA). Moreover, the effects of WEDM machining input parameters on the MRR, surface roughness, gap voltage, gap current, and cutting rate for AISI D2 steel were analyzed by Singh et al. [28]. Taguchi L27 orthogonal array (OA) was also used along with RSM and ANOVA. Furthermore, the effects of WEDM input parameters on the MRR for hot die steel AISI H-11 were explained by Singh et al. [29], wherein input parameters such as pulse-on time, pulse-off time, gap voltage, peak current, wire feed, and wire tension, were studied. The one variable at a time (OVAT) approach showed that pulse-on time was directly proportional to the MRR. Selvakumar et al. [30] studied the effects of WEDM input parameters on cutting speed, surface roughness, and the taper error for AISI D3 tool steel. Taguchi-based grey relational analysis (GRA) was used along with the Taguchi L_9_ OA for the analysis. In another work, the effects of pulse-on time, pulse-off time, wire feed, and wire tension on the MRR and surface roughness of tungsten carbide were studied by Masooth and Arunnath [31]. Again, Taguchi L_9_ OA, along with ANOVA, was used. Moreover, the effects of WEDM input parameters (pulse-on time, pulse-off time, wire feed, flushing pressure, spark voltage, and wire tension) on the surface roughness and the MRR for high-strength armor steel were studied by Bobbili et al. [32] using Taguchi’s L27 OA. Mohamed and Lenin [33] also studied the effects of WEDM input parameters on the machining time for aluminum 6082 T6 alloy using pulse-on time, pulse-off time, and current as the input parameters. Satyanarayana [34] examined the same for Inconel 600. Deshmukh et al. [35] explained the effects of WEDM input parameters on the surface roughness and kerf width of AISI 4140 using pulse-on time, pulse-off time, servo voltage, and wire feed as input parameters. Taguchi’s L_9_ OA was used along with GRA, ANOVA, and regression analysis to analyze the interactions and main effects. For DC53 die steel, Nawaz et al. [36] examined the effects of WEDM input parameters on the MRR, kerf width, and surface roughness. Similarly, Gavisiddesha et al. [37] studied the effects of WEDM input parameters on surface roughness of composite material (AL6061/SICP) using pulse-on time, pulse-off time, and current as input parameters. Again, Taguchi’s L_9_ OA was used along with ANOVA. Input parameters such as pulse-on time, pulse-off time, servo voltage, peak current, wire tension, and water pressure were further used to study the effects of WEDM input parameters on the surface roughness of Vanadis-4E (powder metallurgical cold worked tool steel) by Sudhakara and Prasanthi [38] using design of experiments (DOE). Khan et al. [39] examined the effects of WEDM input parameters on stainless steel’s surface roughness and kerf width (SS 304) using Taguchi’s DOE with L_9_ OA, GRA, and ANOVA. Introducing spark gap as an output parameter in addition to the MRR and surface roughness, Rajyalakshmi and Ramaiah [40] explained the effects of WEDM input parameters (pulse-on time, pulse-off time, voltage, flushing pressure, wire feed rate, wire tension, spark gap, and servo feed) on Inconel 825. Taguchi’s DOE with L36 OA was used along with GRA and ANOVA. Lastly, Lingadurai et al. [41] studied the effects of WEDM input parameters on the metal removal rate, kerf width, and surface roughness of stainless steel AISI grade-304. Again, Taguchi’s DOE with L18 OA was used along with ANOVA. 

In light of the reviewed state-of-the-art, it is evident that optimizing the input process parameters of WEDM is one of the most important design objectives for achieving a higher MRR. Taguchi DOE and ANOVA are the most effective techniques for determining the optimal settings of process variables and their corresponding interaction effects for a given target. Moreover, in previous approaches, the majority of optimization has been conducted on different alloys and super alloys by using different optimization techniques such as optimization through mathematical modeling, full factorial design, optimization through RSM, and the finite element method. Compared to these approaches, Taguchi’s DOE is not only straightforward, efficient, and trustworthy for decreasing costs and enhancing quality, but it also significantly decreases the number of trials. 

Consequently, in this work, the parametric optimization of machining process parameters of WEDM is undertaken with the help of Taguchi DOE and ANOVA, considering AISI 1045 medium carbon steel to increase the MRR. The remainder of the paper is structured as follows: Section 2 discusses the adopted methodology in detail along with experimentation and results; Section 3 discusses the results; and Section 4 concludes the paper with the optimal input parameter settings along with validation. 

## 2. Materials, Methodology, and Experiments

**Figure 1:**
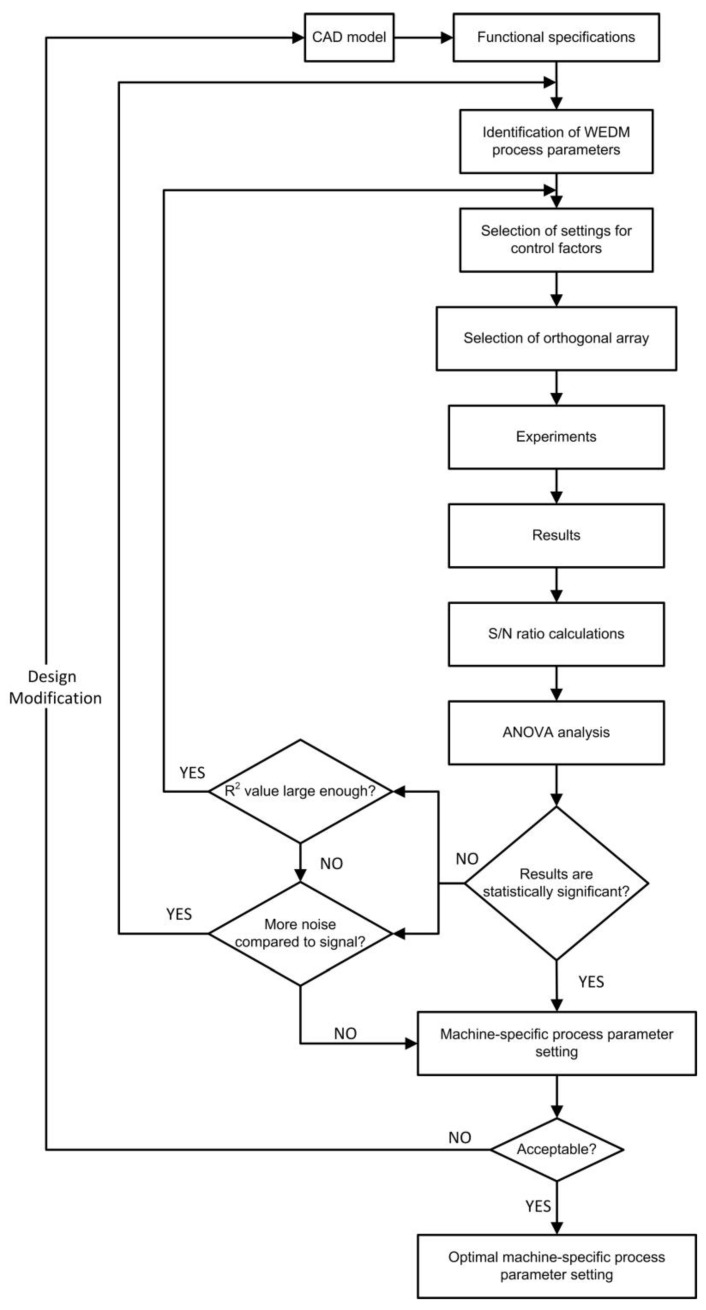
Proposed Methodology for selection of optimal process parameter settings (modified from Zaman et al. [43]).

This paper uses the parametric optimization of process parameters with the help of Taguchi’s DOE and ANOVA, which is an effective way to handle responses triggered by numerous factors. In order to pick the ideal process conditions, robust testing equipment provides a simple, effective, and thorough method. When it comes to correctly modeling response factors, a large number of experiments are no longer necessary with the Taguchi DOE. The foremost objective of the Taguchi method is to optimize the various design criteria. It can also change the process parameters and settings if the results do not meet the functional requirements. The graphical representation of the proposed methodology is shown in Figure 1, which is not only “generic” in nature but can also be used to optimize various design criteria. It further has the flexibility to modify the process parameters and the relevant settings if the results are not as per the functional specifications. The methodology starts from the CAD model, from which the functional specifications are extracted, and control factors are identified for the WEDM process. The rest of the steps in the methodology are explained step by step in the sections to follow.

AISI 1045 medium carbon steel (the country of production of AISI 1045 medium carbon steel is China, and the manufacturer is Shandong Zhong Tong Boda Iron and Steel Co., Ltd., Liaocheng) was used for experiments in this paper because it is a high-solidity steel with excellent weldability, formability, and maximum hardness. AISI 1045 has sway properties in both standardized and molten forms. AISI 1045 medium carbon steel further offers the best formability in a standardized or heat-treated state. Depending on manufacturer recommendations, various operations can be performed on AISI 1045 steel with the appropriate feeds, tool types, and speeds [42]. The chemical and mechanical properties of AISI 1045 medium carbon steel are illustrated in Table 1 and Table 2, respectively.

The application area chosen for this study is the automotive sector, where an AISI 1045 medium carbon-steel-based ‘timing chain sprocket’ was chosen as a case study for which the MRR was to be maximized using WEDM. A suitable material for the timing chain sprocket was chosen with the help of the reviewed literature and the experience of operators, which signified that AISI 1045 medium carbon steel is not only a good material for gears in automobiles but also has a very high material strength.

### 2.1. Determination of Process Parameters and Relevant Settings

In this paper, the most suited input control factors are selected for the work material, which are listed in Table 3. Since not all the parameters influence the MRR, the working parameters (current, voltage, and pulse-on time) that have the most influence were selected using the reviewed literature [19,29,31,32,33,37].

The method then defines the levels of the control factors that need to be optimized. The number of levels for each factor is determined primarily with the help of operators’ experience and, to some extent, from the reviewed literature [29,31,32,33,37]. The levels of process parameters for DOE are listed in Table 4.

### 2.2. Selection of Orthogonal Array (OA)

Based on the control factors and the settings (levels) chosen, a suitable Taguchi’s OA was created. The design of an OA depends upon the number of elements, the magnitude of each factor, and the interconnections between them. The control variables are believed to be independent [44], and it is assumed that there is no interaction between them. Table 5 displays the Taguchi L_9_ orthogonal array used in this experiment.

### 2.3. Experiments

**Figure 2:**
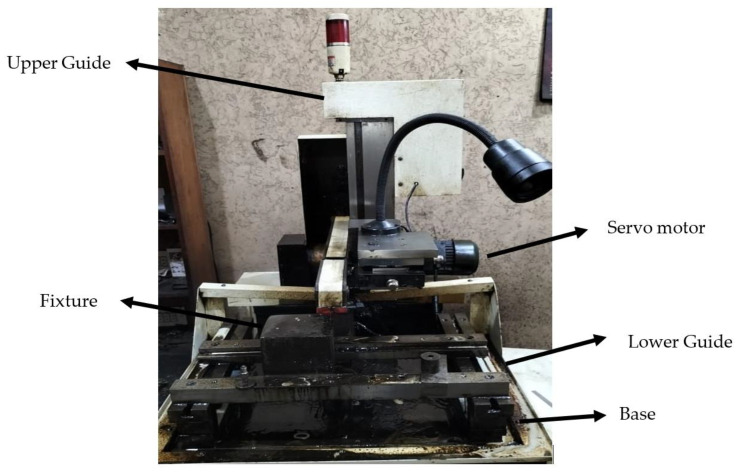
Wire cut electric discharge digital control machine—DK7725.

**Figure 3:**
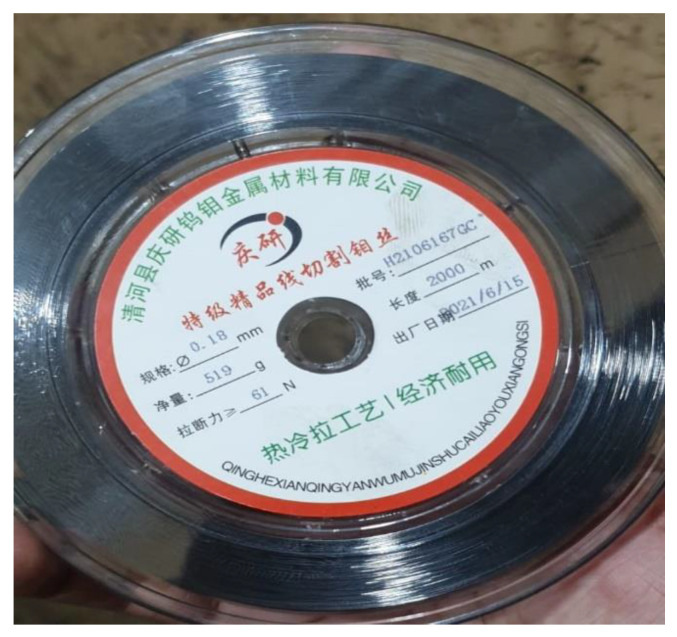
Molybdenum wire.

Tests were carried out on the “DK7725” wire electric discharge machine (the country of manufacture for wire electric discharge machine ‘DK7725′ is China, and the manufacturer is Yinxiang Machinery Manufacturing Co., Ltd., Chongqing, China) (see Figure 2). Molybdenum (the country of manufacture for molybdenum wire is China, and the manufacturer is Dongguan Yingsihai Precision Mold Co., Ltd., Chang’an, China) was used as the electrode wire in the experiment in which AISI 1045 medium carbon steel is used as an anode and molybdenum wire is used as a cathode. A gap was maintained between the tool and the workpiece so that a spark can occur. Deionized water was then used as a dielectric fluid for this experimental setup. The molybdenum wire (Dongguan Yingsihai Precision Mold Co., Ltd., Chang’an, China) used in this experiment is shown in Figure 3, and the specifications of the molybdenum wire are listed in Table 6.

The specifications of the WEDM machine are shown in Table 7, and the dimensions of the timing chain sprocket are listed in Table 8.

**Figure 4:**
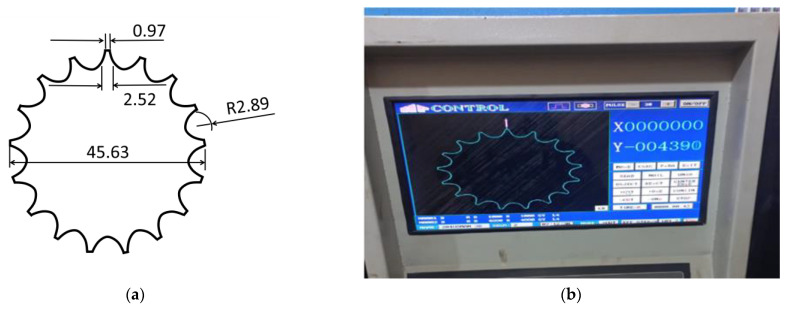
Wire cut electric discharge digital control machining showing (**a**) profile of timing chain sprocket gear teeth (all measurements in mm) and (**b**) the system working on a gear profile.

**Figure 5:**
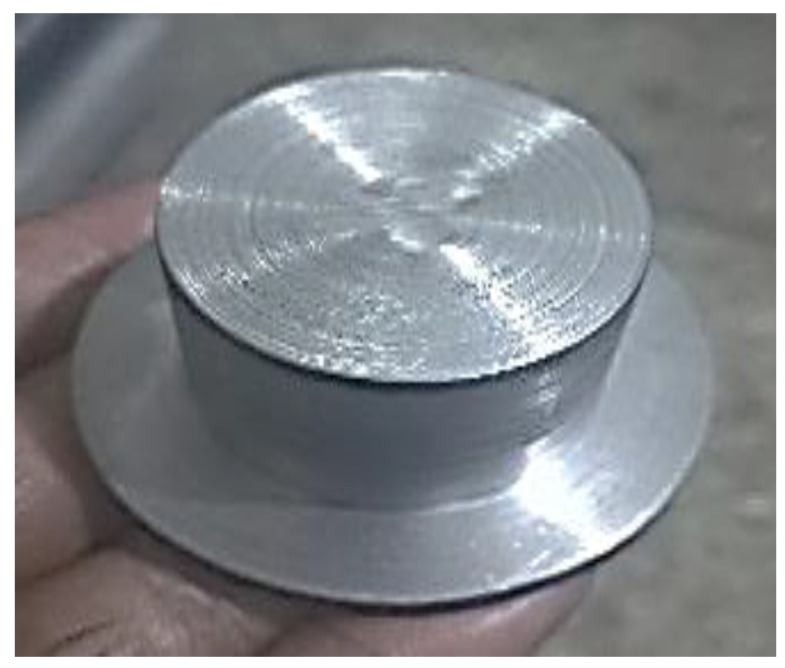
Base of desired part.

**Figure 6:**
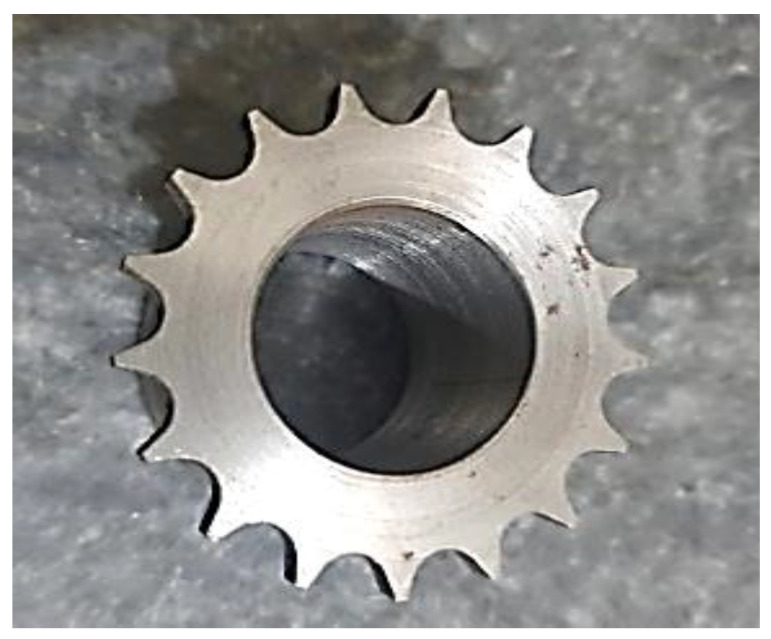
Final part design of timing chain sprocket.

AISI 1045 medium carbon steel is used to manufacture the timing chain sprocket. Based on the chosen L_9_ OA, nine experiments were performed under different levels of controllable factors. First, the design of the timing chain sprocket was made in WEDM-supported computer-aided design (CAD) software (see Figure 4a), which was then imported into the WEDM machine. The different measurements for the profile of the timing chain sprocket gear teeth are also highlighted in Figure 4a. Figure 4b shows the initiation of the WEDM machine process. The DK7725 WEDM uses HF WEDM software (version 8, DESUN machine tool accessories, China) and a control system. The software allows users to customize skim passes, independent lead-ins, lead-outs, glue stops cutting conditions, and four-axis synchronization of complete WEDM programming. As shown in Figure 5, initially, a base part is manufactured, and a gear-cutting operation is then performed on the base part. Figure 6 shows the manufactured timing chain sprocket, which shows that high-quality work is performed with the help of WEDM.

### 2.4. Evaluation of MRR

For the MRR, the material’s density and machining time are taken into consideration [45] using Equation (1) [46]:

(1) $$\mathrm{MRR}\left({\mathrm{mm}}^{3}/\min\right)=\frac{\left[\mathrm{Initial}\;\mathrm{Weight}\;\mathrm{of}\;\mathrm{workpiece}\left(g\right)-\mathrm{Final}\;\mathrm{Weight}\;\mathrm{of}\;\mathrm{Workpiece}\left(g\right)\right]}{\mathrm{Density}\left(g/{\mathrm{mm}}^{3}\right)\times \mathrm{Machining}\;\mathrm{Time}\;\left(\min\right)}$$

The MRRs obtained for the nine experiments performed on WEDM are listed in Table 9. Each MRR obtained is against the control parameters and their associated levels listed in Table 5. For instance, for Experiment 2, for a pulse-on-time of 75 µs, current of 12 A, and voltage of 55 V, the MRR obtained is 0.3165 mm^3^/min for the timing chain sprocket. All of the obtained MRRs would then be analyzed in subsequent sections using the S/N ratio and ANOVA.

### 2.5. Calculation of Signal-to-Noise (S/N) Ratio and Analysis of Variance (ANOVA)

To determine the robustness of a design, the S/N ratio is popularly used where ‘signal’ represents the desired value (MRR) and ‘noise’ shows the undesirable value. Experimentation is the only way to control noise while manufacturing a product. Moreover, in the case of Taguchi DOE, it is important to control the noise variables to generate variability and identify appropriate control element settings that can withstand the effects of disturbance. Equation (2) [43] is used to calculate the S/N ratio. Table 10 shows the S/N ratios:

(2) $$\eta =-10\log_{10}\frac{1}{n}(\sum_{i=1}^{n}\frac{1}{y_{i}^{2}})$$

where *η* is the average S/N ratio, *n* is the number of experiments conducted at level *i*, and *y_i_* is the measured value. As the objective is to maximize the MRR, the ‘larger-the-better’ characteristic is used.

Moreover, ANOVA is a statistical method which tests for differences in means and informs if the means of three or more independent groups vary statistically. In ANOVA, ‘*p*-value’ is used, which can help to determine the effect of responses and the percentage contribution of the error [43].

## 3. Results and Discussion

This research performed nine experiments (using L_9_ OA) with different input controllable factors on an AISI 1045 medium carbon-steel-based timing chain sprocket. The Taguchi DOE was used to find the best input factor settings to obtain the maximum MRR. Thereafter, the S/N ratio and ANOVA were applied. The results from the S/N ratio are shown in Table 10, and their ranking is shown in Table 11. Table 11 clearly shows that the current ranks first, indicating that the current has the greatest influence on the MRR. The results from the ANOVA are also listed in Table 12 for the MRR vs. current, voltage, and pulse-on time. It is evident from Table 12 that the MRR vs. the current shows that the concerned p-value is 0.026, thereby implying that the current has a statistically significant impact on the MRR.

Moreover, the R^2^ value for the ANOVA model for the MRR was 70.23% for the effect of current. For the other two process parameters (voltage and pulse-on time), the R^2^ values were not significantly higher.

**Figure 7:**
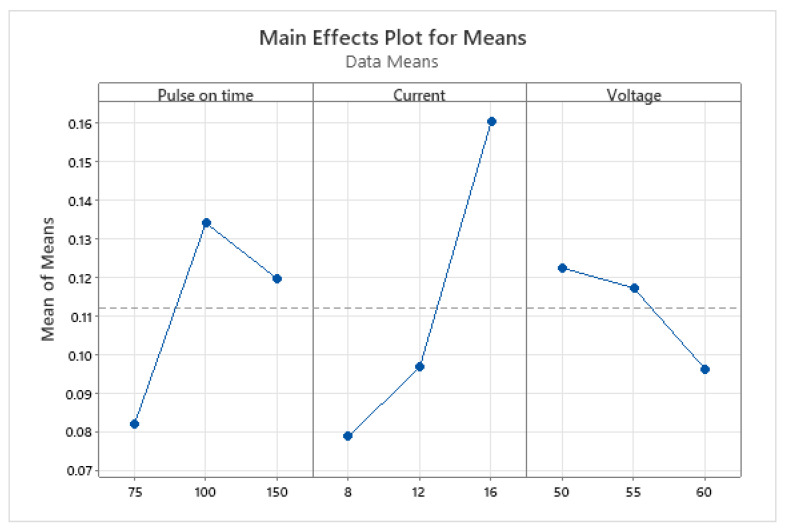
Main effects plot for means (pulse-on time is in µs, current in amperes, and voltage in volts).

**Figure 8:**
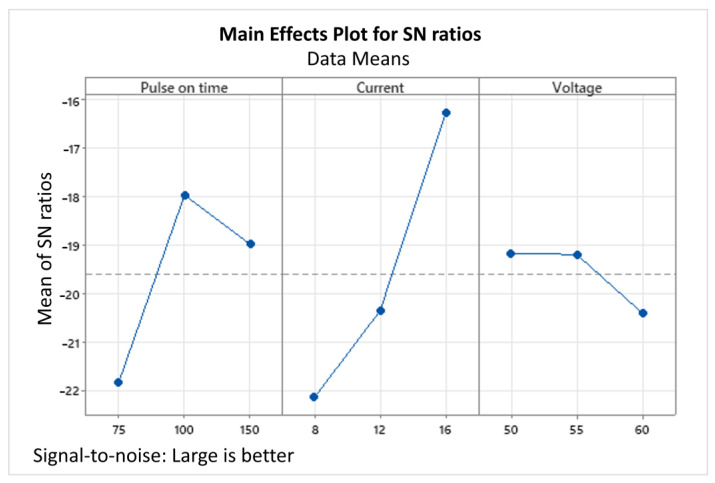
Main effects plot for S/N ratios (pulse-on time is in µs, current in amperes, and voltage in volts).

Figure 7 and Figure 8 show the main effect plot for means and S/N ratios. Thus, a combined analysis of Table 11 and Figure 7 and Figure 8 shows that I = 16-amp, V = 50-volt, and pulse-on time = 100 µs were observed as the optimum parameters to reach the maximum MRR for the timing chain sprocket. A confirmation run was also conducted using the optimal set, which was approved by the automobile manufacturing industry.

The results of the study showed that the current had the greatest influence as an input parameter on the MRR for AISI 1045 medium carbon steel, followed by pulse-on time and voltage. As the current was increased, the MRR also increased. However, when the voltage was increased, the MRR decreased. The impact of increased current for increased MRR in the case of AISI 1045 medium carbon steel was also supported by Mohammadumar et al. [42] and Patel et al. [47]. This is because the voltage determines the width of the spark gap between the leading edge of the electrode and the workpiece. Higher voltage settings increase the gap, due to which the number of sparks decreases, and the machining rate slows down. Similarly, as the pulse-on time increased, the MRR decreased. Material removal is directly proportional to the amount of energy applied during the pulse-on time. This energy is controlled by the peak current and the length of the pulse-on time. However, the extreme pulse duration can be counterproductive. When the optimum pulse duration for each tool and workpiece material combination was exceeded, the MRR started to decrease. Long pulse durations can also restrict the electrodes from machining.

## 4. Conclusions

Optimizing the input process parameters of WEDM is one of the most important design objectives for achieving a greater MRR. Taguchi DOE and ANOVA are the most effective and popular techniques for determining the optimal values of process variables and their corresponding interaction effects for a given target. The Taguchi method is not only straightforward, efficient, and trustworthy for decreasing costs and enhancing quality, but it also significantly decreases the number of trials. This study aimed to find the optimal values for input machine parameters that can maximize the MRR for an AISI 1045 medium carbon-steel-based timing chain sprocket with the help of ANOVA and the S/N ratio.

The optimal values were current = 16 A, voltage = 50 V, and pulse-on time = 100 µs. Furthermore, the methodology adopted in this research has the flexibility to re-design the parts easily if the specified process parameters do not fulfill the functional requirements. Future research can take place along the following lines:

- Experiments can be carried out using multi-objective optimization techniques to find the effect of input parameters on various response factors such as surface roughness and tool wear rate for AISI 1045 medium carbon steel.
- More WEDM parameters can be used, such as wire tension, wire speed, pulse-off time, etc., to understand their effect on MRRs.
- Different grades of steel can be used to make timing chain sprocket gears with different input and output parameters.

## Figures and Tables

**Table 1:** Chemical properties for 1045 medium carbon steel [43].

Component	Percentage (%)
Carbon (C)	0.420–0.50
Iron (Fe)	98.51–98.98
Manganese (Mn)	0.60–0.90
Phosphorous (P)	≤0.040
Sulphur (S)	≤0.050

**Table 2:** Mechanical properties for AISI 1045 medium carbon steel [44].

Mechanical Property	Metric (Unit)
Brinell Hardness (BH)	163
Rockwell Hardness (HR)	84
Ultimate Tensile strength	565 (MPa)
Yield Strength	310 (MPa)
Modulus of Elasticity (E)	200 (GPa)
Bulk Modulus (K)	140 (GPa)
Poisson Ratio (v)	0.290
Shear Modulus (G)	80 (GPa)

**Table 3:** WEDM controllable process parameters.

Ser No.	Control Parameter	Unit
1	Current	Amperes
2	Voltage	Volts
3	Pulse-on time	µs

**Table 4:** Levels of process parameters for DOE.

Symbol	WEDM Machining Parameters	Unit	Level 1	Level 2	Level 3
I	Current	Amp	8	12	16
V	Voltage	Volt	50	55	60
Ton	Pulse-on time	µs	75	100	150

**Table 5:** L_9_ orthogonal array.

Experiment No.	Pulse-On Time (µs)	Current (A)	Voltage (V)
1	75	8	50
2	75	12	55
3	75	16	60
4	100	8	55
5	100	12	60
6	100	16	50
7	150	8	60
8	150	12	50
9	150	16	55

**Table 6:** Specifications of molybdenum wire.

S/No.	Parameters	Unit	Value
1	Diameter of wire	mm	0.18
2	Weight of Roll of wire	g	519
3	Length of wire	m	2000

**Table 7:** WEDM machine (DK 7725) specifications.

Specifications	Value (Unit)
X–Y direction movement	(250 × 320) mm
Table Size	(380 × 525) mm
Thickness of Maximum cut	300 mm or 500 mm
Cut taper/thickness	±3 or ±30/100 mm
Cut accuracy	≤0.015 mm
Wire frame	Adjustable
Maximum endurable load of table	300 kg
Weight of machine	1600 kg
Dimension (L × W × H)	(1450 × 1100 × 1600) mm

**Table 8:** Dimensions of timing chain sprocket.

Ser No.	Parameters	Unit	Values
1	Outer diameter	mm	35.45
2	Height	m	30.8
3	Internal diameter	mm	24
4	Step diameter	mm	32
5	Step length	mm	6

**Table 9:** MRR for AISI 1045 medium carbon steel.

Experiment	MRR (mm^3^/min)
1	0.211
2	0.3165
3	0.3798
4	0.221
5	0.411
6	0.7112
7	0.2421
8	0.3893
9	0.5123

**Table 10:** Response for the ‘larger-the-better’ S/N ratio.

Pulse-On Time (µs)	Current (A)	Voltage (V)	MRR (mm^3^/min)	S/N Ratio
75	8	50	0.211	−13.5144
75	12	55	0.3165	−9.9925
75	16	60	0.3798	−8.4089
100	8	55	0.221	−13.1122
100	12	60	0.411	−7.732
100	16	50	0.7112	−2.9602
150	8	60	0.2421	−12.3201
150	12	50	0.3893	−8.1943
150	16	655	0.5123	−5.8095

**Table 11:** Ranking of input factors.

Level	Pulse-On Time (µs)	Current (A)	Voltage (V)
1	−10.639	−12.982	−8.223
2	−7.932	−8.637	−9.638
3	−8.775	−5.726	−9.484
Delta	2.707	7.256	1.415
Rank	2	1	3

**Table 12:** ANOVA for Source vs. MRR.

Source	Dof	Adj SS	Adj MS	F-Ratio	*p*-Value	% Contribution	Remarks
Current	2	0.14401	0.07200	7.08	0.026 *	70.23	Significant
Error	6	0.06105	0.01018			29.77	
Total	8					100	
Voltage	2	0.01627	0.008133	0.26	0.780	7.93	Insignificant
Error	6	0.18880	0.031466			92.07	
Total	8					100	
Pulse-on time	2	0.03174	0.01587	0.55	0.604	15.48	Insignificant
Error	6	0.17322	0.02899			84.52	
Total	8					100	

* Source of variance with *p*-value less than 0.05 is significant; Dof = degrees of freedom; Adj SS = Adjusted sum of square; Adj MS = Adjusted Mean of square.

## Data Availability

Not Applicable.
